# Interaction strength in plant-pollinator networks: Are we using the right measure?

**DOI:** 10.1371/journal.pone.0225930

**Published:** 2019-12-12

**Authors:** Roberto Novella-Fernandez, Anselm Rodrigo, Xavier Arnan, Jordi Bosch

**Affiliations:** 1 School of Biological Sciences, University of Southampton, Southampton, England, United Kingdom; 2 Universitat Autònoma Barcelona, Cerdanyola del Vallès, Catalunya, Spain; 3 CREAF, Cerdanyola del Vallès, Catalunya, Spain; Universidade Federal de Uberlândia, BRAZIL

## Abstract

Understanding how ecological networks are assembled is important because network structure reflects ecosystem functioning and stability. Quantitative network analysis incorporates measures of interaction strength as an estimate of the magnitude of the effect of interaction partners on one another. Most plant-pollinator network studies use frequency of interaction between individual pollinators and individual plants (encounter) as a surrogate of interaction strength. However, the number of flowers visited per encounter may strongly vary among pollinator and plant species, and therefore not all encounters are quantitatively equivalent. We sampled plant-pollinator interactions in a Mediterranean scrubland and tested whether using a measure of interaction strength based on the number of flowers visited resulted in changes in species (species strength, interaction species asymmetry, specialization) and network descriptors (nestedness, H2’, interaction evenness, plant generality, pollinator generality) compared to the encounter-based measure. Several species (including some of the most abundant ones) showed important changes in species descriptors, notably in specialization. These changes were especially important in plant species with large floral displays, which became less specialized with the visit-based measure of interaction strength. At the network level we found significant changes in all properties analysed. With the encounter-based approach plant generality was much higher than pollinator generality (high specialization asymmetry between trophic levels). However, with the visit-based approach plant generality was greatly reduced so that plants and pollinators had similar levels of generalization. Interaction evenness also decreased strongly with the visit-based approach. We conclude that accounting for the number of flowers visited per encounter provides a more ecologically relevant measure of interaction strength. Our results have important implications for the stability of pollination networks and the evolution of plant-pollinator interactions. The use of a visit-based approach is especially important in studies relating interaction network structure and ecosystem function (pollination and/or exploitation of floral resources).

## Introduction

One of the main aims of community ecology is to understand how interactions among species affect the structure, composition and distribution of biological communities [1]. This task may be complex, as communities are typically composed of tens or hundreds of species in each trophic level, and the number of potential interactions among them increases exponentially with the number of species. As empirical information on trophic and mutualistic relationships has accumulated over the last decades, network analysis has emerged as a useful approach to deal with the complexity of interaction networks (e.g. [2–4]).

Interaction networks are composed of nodes (usually representing species) connected by links (representing interactions). Understanding how these networks are built is important because network structure reflects co-evolutionary processes [5], ecosystem functioning [6], and community stability [7–9]. Therefore, understanding network structure allows to predict ecosystem responses to changes in community structure (e.g. incorporation of new species [10,11] and extinctions [12]), as well as responses to disturbances (e.g. changes in land use [13–15] and climate change [16,17].

The use of network analysis to describe plant-pollinator interactions has rapidly evolved. Initial qualitative or binary networks reflecting the presence/absence of each potential interaction [18] have been replaced by quantitative networks, in which the link among species contains an estimation of the magnitude of the effect of species on one another (interaction strength; [19]. Since the first quantitative plant-pollinator network [20], a series of quantitative descriptors reflecting different aspects of network structure have been developed, including quantitative nestedness [21], network generalization-specialization [22,23], and interaction evenness (a measure of the uniformity of interaction strengths; [13]. Quantitative species descriptors have also been developed to describe the role of each species within the network. These include several specialization indexes [24], as well as indexes of species strength that measure the effect of a species on its partners (or how much the partners depend on this species) [8].

All the above metrics rely on interaction strength. Therefore, its estimation may strongly influence our perception of network structure and species roles. A good measure of interaction strength should reflect the effect of one species on the fitness of its interaction partner. This effect can be decomposed into a quantitative component that describes how frequently two species interact, and a qualitative component that describes the mutual per-encounter effect of the two species [25–27]. In plant-pollinator interactions, the qualitative component can be estimated by measuring the number of pollen grains deposited on the stigmatic surface of the flower and the amount of pollen collected per visit by the pollinator. However, due to the complexity to empirically measure the qualitative component of all interactions in a network, interaction frequency alone is usually used as a surrogate of interaction strength. Different pollinator species deposit different amounts of pollen on the stigmas and remove different amounts of pollen/nectar per-visit (e.g. [25, 28–31]). However, some studies have shown that, at the population level, the impact of the qualitative component is often irrelevant and interaction frequency alone provides a good estimate of the effect of one species on another [27,32,33]. In most plant-pollinator network studies, interaction frequency is measured as the frequency of encounters between an individual pollinator and an individual plant (review in [34]). However, plant-pollinator interactions occur at the individual flower level, and thus, the trophic and reproductive interaction effects of one species on the other will be greater for encounters involving many flower visits. The number of flowers visited per encounter is known to vary across pollinator species [35–37] and to increase linearly with plant floral display (number of open flowers) within a given plant species (review in [38]). We argue that a more ecologically meaningful measure of interaction strength in plant-pollinator networks should be based on the frequency of flowers visited, and that the use of such a measure may produce important changes in network structure.

In this study, we sample plant-pollinator interactions in a scrubland community. We collect data on encounter frequency and on the number of flowers visited per encounter, and combine these two sources of information to obtain a visit-based measure of interaction frequency. Our aim is to assess whether the use of this visit-based interaction strength results in changes in network structure compared to the commonly used encounter-based measure. We have the following hypotheses: 1) At the plant interspecific level, the number of visits per encounter will increase with floral display; 2) The use of a visit-based measure of interaction strength will result in changes in species descriptors of both plants and pollinators, and, in relation to hypothesis 1, these changes will be greater in plant species with larger floral displays; 3) Because different plant species will be differently affected by the change in interaction strength measure (hypothesis 2), we expect the use of the visit-based measure to affect network structure.

## Methods

### Study area

Field work was conducted in a ~ 1 ha plot located in a Mediterranean scrubland in Garraf Natural Park (Barcelona, NE Spain, coordinates: 41.27°N, 01.54°E), at 340 m above sea level and 1700 m from the coastline. The park’s vegetation is dominated by *Quercus coccifera*, *Pistacia lentiscus*, *Rosmarinus officinalis* and *Thymus vulgaris*. Diputació de Barcelona allowed permission to conduct field research in Garraf Natural Park.

### Terminology

In the plant-pollinator literature, the terms “interaction” and “visit” have been given various meanings. We use the term “interaction” to describe the relationship between a pollinator species and a plant species, the term “encounter” to describe an individual pollinator visiting and individual plant [32], and the term “visit” to describe an individual pollinator visiting an individual flower. Therefore, each interaction includes one or more encounters and each encounter one or more visits.

### Flower abundance and floral display

In March-June 2006, we counted, once a week, the number of open flowers of each plant species in six 50 x 1 m transects forming a grid. Distance between adjacent parallel transects was 20 m. Bloom is virtually arrested in late June in coincidence with the summer drought. From these counts we obtained weekly flower density (flowers/m^2^), and floral display (mean number of open flowers per individual at peak bloom) of each plant species.

### Interaction survey

Interaction surveys were conducted twice a week throughout the same period as flower counts. We surveyed the 19 most abundant entomophilous plant species, representing 99.96% of the flowers counted in the six transects. On each sampling day, we haphazardly selected 5–10 representative individuals of the plant species that were in bloom. These individuals were tagged and the number of open flowers on each of them was counted. Then, tagged plants were observed for 4-minute intervals, during which all encounters (pollinators observed on the flowers) were visually identified or captured. This procedure was repeated 5–6 times throughout the day, from 9:00 am to 6:00 pm. Total sampling time was 107 hours during which we recorded 4249 plant-pollinator encounters.

Most pollinators were identified to species level. However, some closely-related species that cannot be distinguished in the field were grouped within the same morphotype (S1 Table). In addition, male and female bees of the same species were classed as different morphotypes because they collect different flower resources (males: nectar; females: nectar and pollen), which may result in differences in flower choice. For convenience we will refer to pollinator morphotypes as “species”.

From these surveys we obtained a weekly measure of encounter frequency, calculated as the number of encounters observed in a week / number of open flowers surveyed in that week.

### Visits per encounter survey

For each plant-pollinator interaction observed, we gathered data on the number of flowers visited per encounter. To do this, we visually followed individual pollinators and, when they reached a plant, counted the number of flowers (inflorescences in Asteraceae) visited during a 4-minute period. It is important to note that our measure of visits per encounter is limited to 4 minutes, and therefore, for pollinator species spending longer periods of time on a given individual plant (e.g. coleopterans), we may have underestimated the number of visits per encounter. These surveys were conducted in 2006, 2007, 2008, 2011 and 2015.

We gathered 4932 visits-per-encounter observations, corresponding to 294 of the 340 interactions observed (86.4%), and accounting for 99.5% of the encounter frequency of the network. With these data we estimated the mean number of visits-per-encounter for each interaction. For many of these interactions (177, representing 95.1% of the encounter frequency), this estimate was based on ≥ 5 observations. There were 46 interactions (0.52% of the encounter frequency) for which we could not obtain data of visits per encounter. For 36 of these (0.49% of the encounter frequency) we used visits-per-encounter values from a functionally similar pollinator species (usually a species of the same genus and with similar body size) on the same plant species. For 4 additional interactions (0.022% of the encounter frequency) we estimated mean visits-per-encounter using expert criteria (i.e., in plants with large flowers, coleopterans usually visited a single flower per encounter during the 4-minute observation period). Finally, for the remaining 6 interactions, we could not obtain any evidence-based estimate of number of visits per encounter and these interactions were excluded from all analyses.

### Interaction strength

As mentioned, most plant-pollinator quantitative network studies use a measure of interaction strength based on the frequency of encounters recorded between each plant and pollinator species [34]. Because flower abundance at a given site varies widely among plant species and sampling effort is often not correlated to flower abundance, encounter frequency should be weighted by flower abundance [39]. Because flower abundance varies through time, our encounter-based interaction strength between plant species A and pollinator species B was calculated as the sum of weekly encounter frequencies (number of encounters between A and B observed in a week) divided by the number of open flowers of species A surveyed in that week, multiplied by the weekly flower abundance of species A. To obtain visit-based interaction strength, we multiplied the encounter-based strength by the mean number of flower visits per encounter of each interaction.

### Species descriptors

For each plant and pollinator species in the network, we calculated three species descriptors using the two interaction strength measures (encounter- and visit-based). *Species strength*. Provides a quantitative measure of the importance of a species for its partners. It is calculated as the sum of the dependences of all the partners on that species [8]. *Species interaction asymmetry*. A measure of the imbalance between the effect that a species has on its partners and the dependence of that species on their partners. We use the Push-pull index [40], which ranges from -1 to 1. Positive values indicate that a species affects its interaction partners more strongly than it is affected by them (“pusher” species). Negative values indicate that a species experiences strong effects from its interaction partners but does not exert a strong reciprocal effect on them (“puller” species). 0 indicates total dependence symmetry. *Generalization-specialization*. We use the *d’* index [23], which measures the level of specialization of a species accounting for the relative abundance of its partners, ranging from 0 (minimum specialization) to 1 (maximum specialization).

### Network descriptors

We calculated five network descriptors with each of the two measures of interaction strength. *Nestedness*. A measure of the extent to which specialists interact with species that form perfect subsets of the species with which generalists interact. We use the weighted NODF index [21] as a measure of nestedness. *Plant generality*. The weighted mean number of pollinator species per plant species. Equivalent to vulnerability in food webs [22]. *Pollinator generality*. The weighted mean number of plant species per pollinator species [22]. *Network specialization*. We use *H*_*2*_*'* [23], which ranges from 0 (maximum generalization) to 1 (maximum specialization). *Interaction Evenness*. A measure of the uniformity of interaction strengths in the network [13]. All species and network descriptors were calculated with *bipartite* package 2.05 [41] in R v.3.2.4 statistical environment [42].

### Statistical analyses

All statistical analyses were conducted in R.

Objective 1. We used a linear mixed model (function *glmer* in R package *lme4* [43]) to test for differences in number of visits per encounter among plant species, using visits per encounter as response variable and plant species as fixed factor. Each plant-pollinator species interaction was a replicate. Since the number of flowers that a pollinator species visits per encounter might be influenced by the pollinator group (ants, bees, Coleoptera, Diptera, Heteroptera, Lepidoptera, Orthoptera and wasps) [35–37], pollinator group was added as a random factor. We then used a linear regression to test whether plant species with larger floral displays (predictor variable) received more visits per encounter (response variable: mean visits per encounter).

Objective 2. To test whether changing the measure of interaction strength affected plant and pollinator species descriptors, we run a linear regression model for each species descriptor with the encounter-based value as the predictor variable, the visit-based value as the response variable, and species as replicates. A slope not significantly different from 1, in combination with a high coefficient of determination, would indicate that the species descriptor is not substantially affected by the measure of interaction strength. To comply with normality assumptions, species strengths (both encounter- and visit-based) were log-transformed and *d’* specialization index of plants was arcsin (sqrt) transformed. We expected plant species with larger flower displays to be most affected by the change in the measure of interaction strength. Thus, for each plant species descriptor separately, we used linear regression models with change in the descriptor (ratio of the value obtained using the visit-based strength divided by the value obtained using the encounter-based strength) as the response variable, and floral display as the predictor variable. Floral display for all descriptors and ratios of change of species strength were log-transformed. Ratio of change of *d’* specialization index was square root transformed.

Objective 3. To test whether the two interaction strength measures produced differences in network descriptors we used a bootstrapping approach. We randomly subsampled the original pool of observed encounters 100 times to obtain 100 random encounter-based networks. We then used visit-per-encounter data to transform these 100 networks into visit-based networks, thus obtaining 100 pairs of networks. Descriptors of the encounter-based and visit-based networks were then compared with paired *t*-tests. Some network descriptors are sensitive to network size [41]. For this reason, this procedure was repeated three times with different subsampling intensities (25, 50 and 75% of the original number of encounters).

## Results

We recorded 4249 encounters of pollinators with plants. The resulting network comprised 334 interactions between 19 plant and 122 pollinator species (39 bees, 27 Coleoptera, 20 Diptera, 15 Lepidoptera, 9 wasps, 8 ants, 3 Heteroptera and 1 Orthoptera; S1 Table).

**Fig 1:**
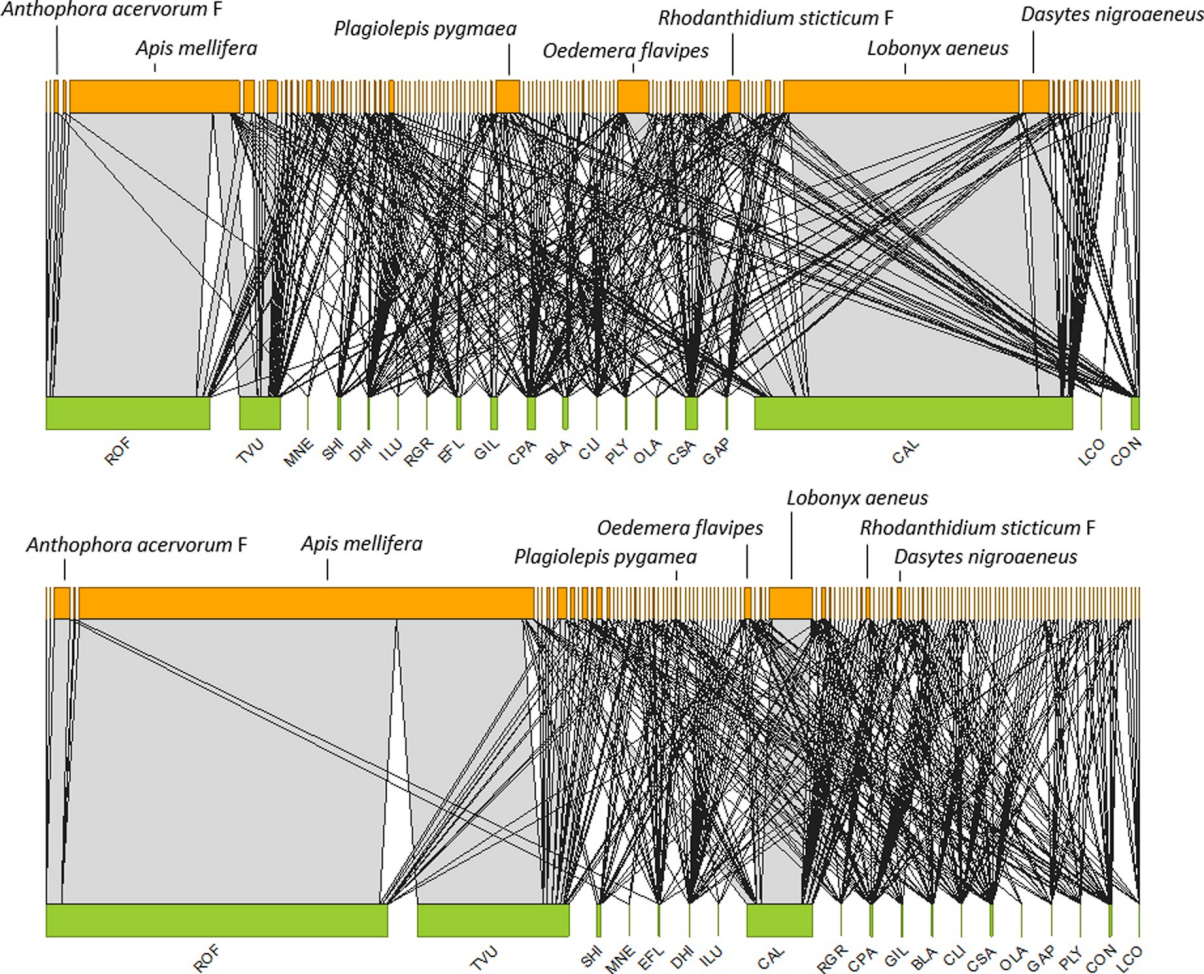
The plant-pollinator network according to encounter-based (top) and visit-based (bottom) measures of interaction strength. Pollinator species in orange, plant species in green. Width of horizonal bars denotes interaction frequency of each species. Gray bar width denotes interaction strength. See Table 1 for plant species.

The encounter-based and the visit-based networks are represented in Fig 1. Both were characterized by a few very strong interactions and a large majority of weak interactions. In both matrices, the plant species with the highest interaction frequency were *Cistus albidus*, *Rosmarinus officinalis* and *Thymus vulgaris*, and the two most-frequently interacting pollinator species were the beetle *Lobonyx aeneus* and the honey bee, *Apis mellifera* (Fig 1). However, the two networks showed marked differences in the interaction strength of the three main interactions. In the visit-based network, the interactions *A*. *mellifera-R*. *officinalis* and *A*. *mellifera-T*. *vulgaris* became much stronger, whereas the interaction *L*. *aeneus-C*. *albidus* became much weaker (Fig 1).

### Visits per encounter (Objective 1)

**Fig 2:**
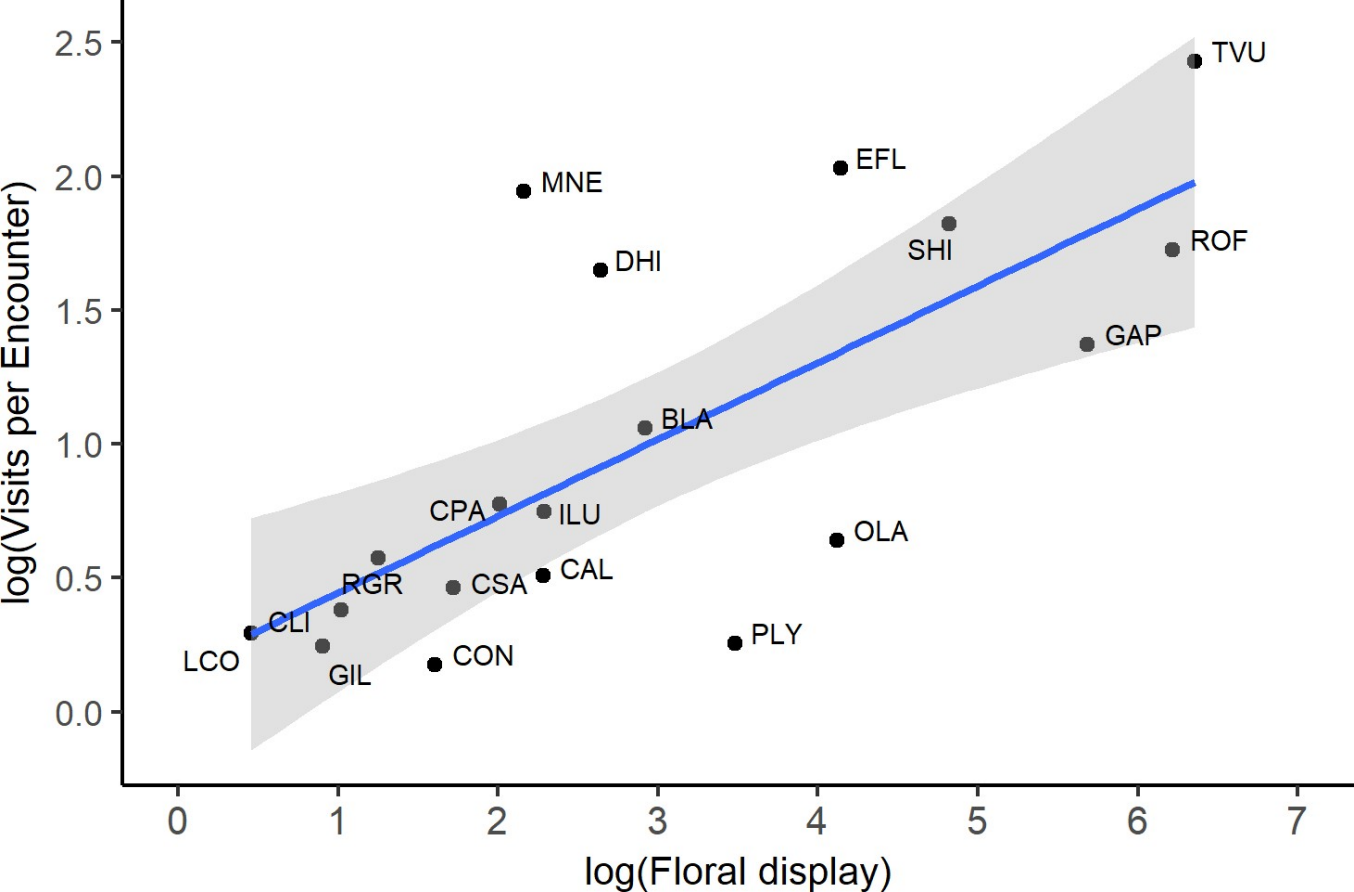
Relationship between mean number of visits per encounter (log-transformed) and floral display (log-transformed) (p = 0.0005; R^2^ = 0.49; n = 19 plant species). Shaded areas denote 95% confidence interval. See Table 1 for plant species names.

Different plant species received different numbers of visits per encounter (Linear mixed model; χ^2^_18_: 291.3; p<0.0001; Table 1), and these differences were positively correlated to floral display (Linear regression model; F_17_ = 18.2, p = 0.0005, R^2^ = 0.49) (Fig 2).

**Table 1:** Number of pollinator species, mean ± SD number of visits per encounter (mean of all pollinator species) and floral display (mean number of open flowers at peak bloom) for each plant species surveyed.

Code	Plant species	Pollinator	Visits per encounter	Floral display
		species (n)		
TVU	*Thymus vulgaris*	19	11.3 ± 9.3	576
EFL	*Euphorbia flavicoma*	17	7.6 ± 7.2	63
MNE	*Muscari neglectum*	3	7.0 ± 3.7	9
SHI	*Sideritis hirsuta*	11	6.2 ± 6.8	124
ROF	*Rosmarinus officinalis*	15	5.6 ± 6.0	502
DHI	*Dorycnium hirsutum*	25	5.2 ± 5.7	14
GAP	*Galium aparine*	16	3.9 ± 2.8	294
BLA	*Biscutella laevigata*	18	2.9 ± 2.5	19
CPA	*Centaurea paniculata*	28	2.2 ± 1.5	7
ILU	*Iris lutescens*	4	2.1 ± 1.4	10
OLA	*Orobanche latisquama*	3	1.9 ± 0.9	62
RGR	*Ranunculus gramineus*	16	1.8 ± 1.2	3
CAL	*Cistus albidus*	22	1.7 ± 1.2	10
CSA	*Cistus salvifolius*	20	1.6 ± 0.9	6
CLI	*Centeurea linifolia*	25	1.5 ± 0.6	3
LCO	*Leuzea conifera*	11	1.3 ± 0.5	2
PLY	*Phlomis lychnitis*	12	1.3 ± 0.4	33
GIL	*Gladiolus illyricus*	8	1.3 ± 0.3	2
CON	*Convolvulus althaeoides*	21	1.2 ± 0.4	5

### Species descriptors (Objective 2)

#### Species strength

**Fig 3:**
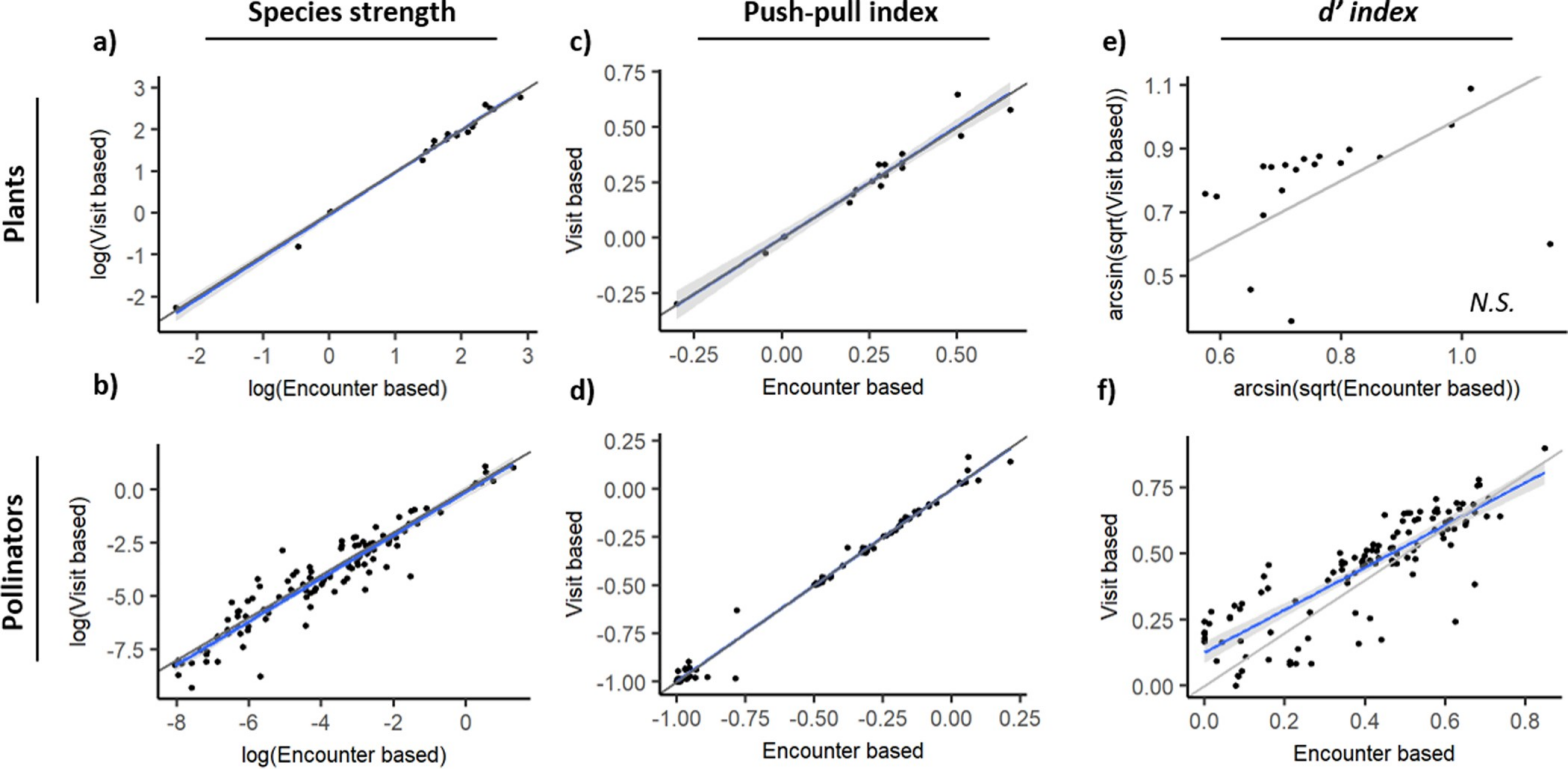
Relationship between encounter-based and visit-based measures of three species descriptors (species strength, push-pull index and *d’* specialization index) for plants (a, c, e) and pollinators (b, d, f). Shaded areas denote 95% confidence intervals. Grey lines indicate slope = 1.

**Fig 4:**
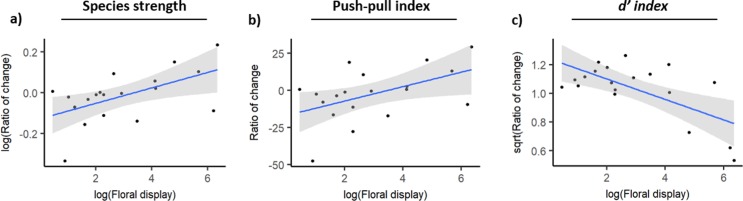
Relationship between the magnitude of the change (ratio of the visit-based value divided by encounter-based value) of three species descriptors (species strength, push-pull index and *d’* specialization index) and floral display of plant species. Shaded areas denote 95% confidence intervals.

S1A Fig shows the strength of each plant species calculated based on the two interaction strength measures. The two measures of plant species strength were highly and positively related, and the slope of their relationship was not different from 1 (Linear regression model: F_17_ = 1861.0, p<0.0001, R^2^ = 0.99, β±SE: 1.01±0.024; Fig 3A), thus indicating that species strength was not substantially affected by the measure of interaction strength used. Nonetheless, the magnitude of change from encounter-based species strength to visit-based species strength increased with floral display (Linear regression model: F_17_ = 7.5, p = 0.014, R^2^ = 0.26, Fig 4A).

The strength of the 30 main pollinator species is shown in S1B Fig. Interestingly, the strongest species (*Plagiolepis pygmaea*, *Rhodanthidium sticticum* M and *Rhodanthidium sticticum* F) were not involved in the strongest interactions (Fig 1). The two measures of interaction strength were again highly and positively correlated, and the slope of their relationship was not different from 1 (F_122_ = 1092.0, p<0.0001, R^2^ = 0.90, β±SE: 1.01±0.031; Fig 3B). However, some of the strongest species experienced important changes when comparing the two interaction strength measures. For instance, *A*. *mellifera* strength increased 1.74-fold, and the strength of *Rhodanthidium sticticum* M and *Plagiolepis pygmaea* decreased 0.71- and 0.76-fold, respectively in the visit-based network (Fig 1).

#### Interaction species asymmetry

We analysed species asymmetry with the push-pull index. Most plant species were "pushers" (had high positive index values; S2A Fig), indicating they had a strong effect on their pollinators but were not highly dependent on them. The values of the push-pull index obtained with the two interaction strength measures were highly and positively correlated, and the slope of this relationship was not different from 1 (F_17_ = 362.6, p<0.0001, R^2^ = 0.95; β±SE: 1.00±0.053; Fig 3C). No species changed from pusher to puller or vice-versa (S2A Fig). However, changes in push-pull index tended to be greater in species with large floral displays (F_17_ = 5.5, p = 0.031, R^2^ = 0.20; Fig 4B). That is, species with greater floral displays increased their effect on pollinators and species with smaller displays became more dependent on pollinators.

Most pollinator species were "pullers", depending strongly on certain plant species but barely affecting them (S2B Fig). The values of the push-pull index obtained with the two interaction strength measures were again highly and positively correlated, and the slope of this relationship was not different from 1 (Linear model: F_122_ = 17000, p<0.0001, R^2^ = 0.99; β±SE: 1.00±0.008; Fig 3D). However, some of the pusher species experienced important changes (e.g. 2.78-fold in *A*. *mellifera*) (S2B Fig). As with plants, no species changed from pusher to puller or vice-versa.

#### Generalization-specialization

Species specialization was characterized with the *d’* index. For plants, specialization values obtained with the two measures of interaction strength were not significantly related (F_17_ = 1.4, p = 0.25; Fig 3E). For most species, specialization increased with the visit-based measure of interaction strength, but three species experienced strong declines (S3A Fig). The magnitude of the change in *d’* decreased with floral display (Linear model: F_17_ = 12.9, p = 0.002, R^2^ = 0.40; Fig 4C). That is, plant species with larger floral displays tended to become more generalized in the visit-based network.

Specialization of pollinator species with the two measures of interaction strength was significantly and positively related, but the slope was significantly different from 1 (Linear model: F_122_ = 306.9, p<0.0001, R^2^ = 0.71; β±SE:0.80±0.046; Fig 3F). As with plants, some species experienced strong (2-3-fold) changes when using the visit-based approach (S3B Fig).

### Network descriptors (Objective 3)

We obtained consistent significant differences between paired encounter-based and visit-based subnetworks for all descriptors across the three subnetwork sizes (Table 2). The magnitude of these differences was always comparable to the magnitude of the differences between the two original networks (Table 2). The magnitude of these changes varied greatly among descriptors (Table 2). Nestedness experienced a moderate increase and *H*_*2*_*'* a very small decrease. On the other hand, interaction evenness decreased markedly. Interestingly, plant generality decreased very strongly, whereas pollinator generality increases slightly. As a result, the large differences in generality between the plant and pollinator trophic levels obtained with the encounter-based measure (3.53 vs 1.88) almost disappeared with the visit-based measure (2.16 vs 2.03) (Table 2).

**Table 2:** Descriptors of the original encounter-based and visit-based networks and of the networks obtained through bootstrapping (random subsamples of 25, 50 and 75% of the original interactions) (mean ± SD, n = 100). P-values of paired *t*-tests comparing encounter- and visit-based subsampled networks are shown.

Network descriptor	Interaction strength measure	Original networks	25% subsample networks		50% subsample networks		75% subsample networks	
			Mean ± SD	P	Mean ± SD	P	Mean ± SD	P
**Nestedness (wNODF)**	Encounter	14.56	12.16 ± 0.99	<0.0001	13.19 ± 0.68	<0.0001	14.04 ± 0.49	<0.0001
	Visit	16.05	13.67 ± 1.16		14.60 ± 0.78		15.42 ± 0.54	
**Interaction evenness**	Encounter	0.32	0.32 ± 0.02	<0.0001	0.32 ± 0.01	<0.0001	0.32 ± 0.01	<0.0001
	Visit	0.22	0.22 ± 0.03		0.22 ± 0.01		0.22 ± 0.01	
**Network Specialization (*H***_***2***_***’*)**	Encounter	0.64	0.69 ± 0.04	0.0081	0.66 ±0.03	0.0009	0.64 ± 0.02	<0.0001
	Visit	0.62	0.68 ± 0.06		0.65 ± 0.04		0.63 ± 0.02	
**Plant generality**	Encounter Visit	3.53	3.06 ± 0.28	<0.0001	3.30 ± 0.17	<0.0001	3.45 ± 0.10	<0.0001
		2.16	1.95 ± 0.25		2.07 ± 0.15		2.13 ± 0.10	
**Pollinator generality**	Encounter	1.88	1.75 ± 0.08	<0.0001	1.81 ± 0.05	<0.0001	1.85 ± 0.03	<0.0001
	Visit	2.03	1.98 ±0.12		1.99 ± 0.08		2.02 ± 0.04	

## Discussion

Understanding how different approaches to estimate interaction strength may influence network structure has generated much debate in the interaction literature (e.g. [22,44–46]). The commonly used measure of interaction strength in plant-pollinator systems is based on the frequency of encounters between individual plants and pollinators. However, flower visits better quantify the number of events in which pollen deposition and/or floral resource uptake may occur, and thus a measure based on frequency of flower visits provides a more ecologically meaningful interaction strength measure. We show that using a visit-based measure results in important changes in interaction strength and network structure.

The use of a visit-based approach is only justified if plant species show significant differences in the number of visits-per-encounter. Thus, our first objective was to establish whether these differences occurred and whether they were related to floral display. Previous studies have shown that, within a plant species, the number of visits per encounter increases linearly with floral display [38,47,48]. At the interspecific level, the situation is more complex for two reasons. First, different plant species differ not only in floral display, but also in the amounts of pollen and nectar produced per flower. Second, different plant species are visited by different pollinators, and, within a plant species, different pollinator species are known to visit different proportions of the available open flowers [35–37]. Our results show that the positive and linear relationship between visits per encounter and floral display is maintained at the community level.

Our second objective was to determine whether the use of a visit-based measure of interaction strength, as opposed to an encounter-based measure, would result in changes in species properties. We analysed changes in species strength, interaction species asymmetry (push-pull index), and specialization (*d’* index). Species strength and interaction species asymmetry values obtained with the two interaction strength measures were highly correlated. On the other hand, specialization values obtained with the two measures were correlated only for pollinators and then with a slope different from 1. Overall, several plant and pollinator species (including some with high interaction frequencies) experienced important changes in one or more of the metrics measured. For example, species strength of the honey bee, *Apis mellifera*, almost doubled, and specialization of *Thymus vulgaris* decreased more than two-fold. As predicted, changes in plant species metrics tended to be greater in species with larger floral displays. Plant species with larger floral displays tended to become less specialized.

Our third objective was to establish whether using a visit-based measure of interaction strength resulted in changes in the network structure. We found significant changes in the five metrics that we analysed. The encounter-based measure produced a strong asymmetry in specialization between the two trophic levels, with pollinators being much more specialized than plants. However, plant generality strongly declined with the visit-based approach, resulting in similar levels of specialization in the two trophic levels. The visit-based approach also produced a strong decline in interaction evenness. These structural changes have important ecological consequences for the stability of plant-pollinator communities. Specialization in pollination networks has been linked, both theoretically and empirically, to susceptibility to disturbances and extinction risk (e.g. [14,49]). Our results also have consequences on how we perceive the evolution of plant-pollinator interactions, as high levels of specialization facilitate the appearance of tight coevolutionary processes [5,50,51]. We conclude that accounting for the number of visits per encounter results in important changes in our perception of plant-pollinator interactions. Such an approach provides a better measure of the number of events in which pollen deposition and/or floral resource uptake may occur, and therefore is particularly relevant in studies exploring the relationship between network structure and function.

## Supporting information

S1 Table Pollinator species (M: male; F: female) of the Garraf community. (DOCX)

S1 Fig Plant (a) and pollinator (b) species strength according to two measures of interaction frequency, an encounter-based measure and a visit-based measure. See Table 1 for plant species names. Only the 30 main pollinator species (based on encounter interaction strength) are represented.
(DOCX)

S2 Fig Plant (a) and pollinator (b) species push-pull index according to two measures of interaction frequency, an encounter-based measure and a visit-based measure. Positive values indicate that a species is a “pusher” (species that affect their interaction partners more strongly than they are affected by them). Negative values indicate that the species is a “puller” (species that experience strong effects from their interaction partners but do not exert a strong reciprocal effect on them). See Table 1 for plant species names. Only the 30 main pollinator species (based on encounter interaction strength) are represented.
(DOCX)

S3 Fig Plant (a) and pollinator (b) specialization (*d’* index) according to two measures of interaction frequency, an encounter-based measure and a visit-based measure. See Table 1 for plant species names. Only the 30 main pollinator species (based on encounter interaction strength) are represented.
(DOCX)

S1 Dataset Plant-pollinator interactions in the network and their interaction strengths using the encounter and visit based measures. (CSV)

S2 Dataset Floral display of the plant species in the network. (CSV)
