# Supplementary material for: Interaction strength in plant-pollinator networks: Are we using the right measure?
Source: PLoS One. 2019 Dec 12;14(12):e0225930. doi: 10.1371/journal.pone.0225930 (PMC6907754; doi:10.1371/journal.pone.0225930)
Supplement: S1 Table — (DOCX) [file pone.0225930.s001.docx]

**Supporting information**

**S1** **Table**

| **Code** | **Pollinator species** | **Pollinator group** |
| --- | --- | --- |
| 1 | *Apis mellifera* | bees |
| 2 | *Andrena angustior F* | bees |
| 3 | *Andrena nigroaenea F* | bees |
| 4 | *Andrena nigroaenea M* | bees |
| 5 | *Andrena rhenana F* | bees |
| 6 | *Andrena senecionis M* | bees |
| 7 | *Anthidiellum strigatum F* | bees |
| 8 | *Anthidium cingulatum F* | bees |
| 9 | *Anthophora acervorum F* | bees |
| 10 | *Anthophora crassipes M* | bees |
| 11 | *Anthophora dispar F* | bees |
| 12 | *Anthophora dispar M* | bees |
| 13 | *Bombus terrestris F* | bees |
| 14 | *Ceratina cucurbitina F* | bees |
| 15 | *Ceratina cyanea F* | bees |
| 16 | *Colletes nigricans M* | bees |
| 17 | *Eucera hispaliensis F* | bees |
| 18 | *Eucera nigrescens F* | bees |
| 19 | *Hoplitis acuticornis F* | bees |
| 20 | *Hylaeus hyalinatus F* | bees |
| 21 | *Hylaeus hyalinatus M* | bees |
| 22 | *Lasioglossum albocinctum F* | bees |
| 23 | *Lasioglossum bimaculatum F* | bees |
| 24 | *Lasioglossum griseolum F* | bees |
| 25 | *Lasioglossum griseolum M* | bees |
| 26 | *Megachile apicalis F* | bees |
| 27 | *Megachile pilidens F* | bees |
| 28 | *Megachile pilidens M* | bees |
| 29 | *Megachile pirenaica F* | bees |
| 30 | *Osmia gallarum F* | bees |
| 31 | *Osmia leaiana F* | bees |
| 32 | *Osmia leaiana M* | bees |
| 33 | *Osmia melanogaster M* | bees |
| 34 | *Osmia rufohirta F* | bees |
| 35 | *Osmia submicans F* | bees |
| 36 | *Osmia tricornis F* | bees |
| 37 | *Protosmia exenterata F* | bees |
| 38 | *Rhodanthidium septemdentatum F* | bees |
| 39 | *Rhodanthidium sticticum M* | bees |
| 40 | *Acmaeodera cylindrica* | Coleoptera |
| 41 | *Acmaeoderella adspersula* | Coleoptera |
| 42 | *Acmaeoderella moroderi* | Coleoptera |
| 43 | *Anaspis sp.* | Coleoptera |
| 44 | *Anthaxia millefolii* | Coleoptera |
| 45 | *Anthaxia scutellaris* | Coleoptera |
| 46 | *Anthaxia spinolae* | Coleoptera |
| 47 | *Apion sp.* | Coleoptera |
| 48 | *Aplocnemus calidus* | Coleoptera |
| 49 | *Attalus pictus* | Coleoptera |
| 50 | *Bruchidius spp.* | Coleoptera |
| 51 | *Clytus rhamni* | Coleoptera |
| 52 | *Cryptocephalus ramburi* | Coleoptera |
| 53 | *Dasytes nigroaeneus* | Coleoptera |
| 54 | *Hypera venusta* | Coleoptera |
| 55 | *Lobonyx aeneus* | Coleoptera |
| 56 | *Melighetes spp.* | Coleoptera |
| 57 | *Mordellistena sp. 1* | Coleoptera |
| 58 | *Mordellistena sp. 4* | Coleoptera |
| 59 | *Oedemera sp. A* | Coleoptera |
| 60 | *Oedemera barbara* | Coleoptera |
| 61 | *Oedemera flavipes* | Coleoptera |
| 62 | *Oxythyrea funesta* | Coleoptera |
| 63 | *Psilothrix viridicoerulea* | Coleoptera |
| 64 | *Rhinocyllus conicus* | Coleoptera |
| 65 | *Spermophagus spp.* | Coleoptera |
| 66 | *Stenurella melanura* | Coleoptera |
| 67 | *Bombylius major* | Diptera |
| 68 | *Bombylius discolor* | Diptera |
| 69 | Calliphoridae spp. | Diptera |
| 70 | *Chrysotoxum spp.* | Diptera |
| 71 | *Coenia ?* | Diptera |
| 72 | *Diptera 10* | Diptera |
| 73 | *Eristalinus aeneus* | Diptera |
| 74 | *Eristalis tenax* | Diptera |
| 75 | *Eumerus argyropus* | Diptera |
| 76 | *Eupeodes corollae* | Diptera |
| 77 | *Merodon funestus* | Diptera |
| 78 | *Platynochaetus setosus* | Diptera |
| 79 | *Sarcophagidae sp. 1* | Diptera |
| 80 | *Sarcophagidae sp. 10* | Diptera |
| 81 | *Sarcophagidae sp. 16* | Diptera |
| 82 | *Sarcophagidae sp. 8* | Diptera |
| 83 | *Scaeva spp.* | Diptera |
| 84 | *Sphaerophoria scripta* | Diptera |
| 85 | *Villa aff. hottentota* | Diptera |
| 86 | *Xanthogramma pedissequum* | Diptera |
| 87 | *Camponotus cruentatus* | ants |
| 88 | *Camponotus lateralis* | ants |
| 89 | *Crematogaster sordidula* | ants |
| 90 | *Formica gerardi* | ants |
| 91 | *Formica subrufa* | ants |
| 92 | *Plagiolepis pygmaea* | ants |
| 93 | *Temnothorax niger* | ants |
| 94 | *Temnothorax rabaudi* | ants |
| 95 | *Beosus maritimus* | Heteroptera |
| 96 | *Hadrodemus m-flavum* | Heteroptera |
| 97 | *Lygus italicus* | Heteroptera |
| 98 | *Adelidae spp.* | Lepidoptera |
| 99 | *Anthocaris euphenoides* | Lepidoptera |
| 100 | *Euchloe crameri* | Lepidoptera |
| 101 | *Gonepteryx cleopatra* | Lepidoptera |
| 102 | *Lasiommata megera* | Lepidoptera |
| 103 | *Melanargia occitanica* | Lepidoptera |
| 104 | Microlepidopteran | Lepidoptera |
| 105 | *Minoa murinata* | Lepidoptera |
| 106 | *Pieris brassicae* | Lepidoptera |
| 107 | *Pieris rapae* | Lepidoptera |
| 108 | *Pseudophilotes panoptes* | Lepidoptera |
| 109 | *Pyronia bathseba* | Lepidoptera |
| 110 | *Vanessa cardui* | Lepidoptera |
| 111 | *Zerynthia rumina* | Lepidoptera |
| 112 | *Zygaena lavandulae* | Lepidoptera |
| 113 | Acrididae | Orthoptera |
| 114 | *Braconidae sp. 1* | wasps |
| 115 | *Braconidae sp. 2* | wasps |
| 116 | *Chalcidoidea sp. 1* | wasps |
| 117 | *Chalcidoidea sp. 2* | wasps |
| 118 | *Gasteruption sp.* | wasps |
| 119 | Ichneumonidae | wasps |
| 120 | *Polistes dominulus* | wasps |
| 121 | *Anoplius sp.* | wasps |
| 122 | *Symphita sp. A* | wasps |
